# The first Vietnamese patient who presented late onset of pantothenate kinase-associated neurodegeneration diagnosed by whole exome sequencing: A case report

**DOI:** 10.1097/MD.0000000000034853

**Published:** 2023-10-27

**Authors:** Van Khanh Tran, Chi Dung Vu, Hai Anh Tran, Nguyen Thi Kim Lien, Nguyen Van Tung, Nguyen Ngoc Lan, Huy Thinh Tran, Nguyen Huy Hoang

**Affiliations:** a Hanoi Medical University, Hanoi, Vietnam; b The Center of Endocrinology, Metabolism, Genetics, and Molecular Therapy, Vietnam National Children’s Hospital, Hanoi, Vietnam; c Institute of Genome Research, Vietnam Academy of Science and Technology, Hanoi, Vietnam; d Graduate University of Science and Technology, Vietnam Academy of Science and Technology, Hanoi, Vietnam.

**Keywords:** NBIA, *PANK2* mutation, PKAN, Vietnamese patient, whole exome sequencing (WES)

## Abstract

**Rationale:**

Pantothenate kinase-associated neurodegeneration (PKAN), also called Hallervorden-Spatz syndrome, is a rare autosomal recessive disease associated with brain iron accumulation and characterized by progressive dystonia, dementia, and dysarthria symptoms. PKAN, caused by a defective pantothenate kinase 2 (*PANK2*) gene, is the most common neurodegeneration with a brain iron accumulation (NBIA) group. The “eye of the tiger” sign in the magnetic resonance imaging demonstrated a bilateral hyperintense signal in the basal ganglia region on T2-weighted images, which is a characteristic feature of the diagnosis. PKAN is classified into 2 main types. The early-onset type (classic type) with rapid progression is characterized by symptoms of gait impairment and dystonia leading to loss of ambulation in early childhood. In the later-onset type (atypical type), slow progression usually takes place in the second decade of life with symptoms of neurodegeneration, dystonia, dysarthria, rigidity, choreoathetosis, and motor impairment. Until now, PKAN patients have only been reported in a few countries in Asia such as China, Korea, India, Iran, Taiwan, and Thailand.

**Patient concerns:**

Here we report the first case of PKAN in Vietnam. The patient had a late onset but the disease progresses rapidly with symptoms of dyskinesia, dysphagia, and difficulty speaking.

**Diagnoses:**

Pantothenate kinase-associated neurodegeneration.

**Interventions:**

Whole exome sequencing was performed to identify heterozygous mutations in the *PANK2* gene (NM_153638.4) (c.856C>T, p.Arg286Cys and c.1351C>T, p.Arg451Ter) that has been confirmed as the cause of the disease.

**Outcomes:**

In this study, the first Vietnamese patient with late-onset PKAN was diagnosed by the whole exome sequencing method.

**Lessons:**

The patient’s case marks an important milestone for the first case in Vietnam. The results of the study will provide a scientific basis for clinicians in the diagnosis and genetic counseling of patients.

## 1. Introduction

Pantothenate kinase-associated neurodegeneration (PKAN, OMIM#234200) is the most common type of neurodegeneration with brain iron accumulation (NBIA) group. NBIA is a group of heterogeneous neurodegenerative disorders including symptoms such as postural disturbances (extrapyramidal), abnormal motor, gait impairment, visual impairment, dystonia, dysarthria, and neurodegeneration. Neurodegeneration is detected in specific regions of the central nervous system leading to abnormal progressive involuntary motor symptoms, loss of walking ability, mental confusion, intellectual disability, joint stiffness, and retinitis pigmentosa. NBIA is characterized by radiographic evidence of iron accumulation in the brain and typically begins in childhood. NBIA is classified into separate disorders based on genetic causes and related genes such as the pantothenate kinase 2 (*PANK2*)*, PLA2G6*, and *FA2H*.^[1]^ PKAN (also known as NBIA-1, accounting for nearly 30–50% of NBIA cases all over the world) is a rare genetic disorder characterized by iron deposition in the basal ganglia region and has early onset before age 10.

The incidence of PKAN is estimated at 1 to 3: 1000,000.^[2]^ PKAN is divided into 2 main forms: The early-onset form is often rapidly progressive and is characterized by gait impairment and dystonia leading to loss of mobility in adolescence. Most of these children also developed retinopathy (in 2-thirds of patients) and increased deep tendon reflexes (in one-quarter of patients), whereas a few (about 8%) had acanthocytes and aberrant plasma lipoproteins. The classic form has a mean age of onset of 3 to 4 years with symptoms of postural disturbances, progressive extrapyramidal, and corticospinal tract symptoms. Most of these patients develop motor impairment between the age of 5 and 8 and loss of ambulation within 10 to 15 years.^[3]^ Extrapyramidal symptoms, dystonia, dysarthria, stiffness, neurological manifestations, attention deficit hyperactivity disorder, chorea, Parkinson, and cortical symptoms have also been reported in these patients.^[4]^

Later-onset atypical form (10 years of age) with symptoms of speech and mental disorders, dystonia, spasticity, increased deep tendon reflexes, and chorea (accounting for 25% of patients).^[4]^ Neurobehavioral abnormalities, psychosis, intellectual disability, cortical symptoms, gait impairment, and extrapyramidal symptoms were also observed in these patients.^[4]^ In these patients, dystonia was the most common symptom (87%), in which the muscles of the upper and lower limb muscles extremities were affected leading to axial dystonia, difficulty walking, tremors, and chorea. Other symptoms such as spasticity, increased deep tendon reflexes, and plantar extensor reactions occurred in 25% of patients and cognitive impairment was detected in 29% of patients.^[3]^ Visual impairment and retinal degeneration could be present with less frequency in these patients. The atypical form shows a slower progression than the classical form. The ability to walk could be lost in time from 15 to 40 years after appearing the first symptom.

Until now, there is no specific treatment for PKAN. Treatment requires a combination of specialties including neurologist, ophthalmologist, physiotherapist, and speech therapist.^[4]^ The clinical feature of neurodegeneration due to iron accumulation in the globus pallidus resembling the “eye of the tiger” sign on the magnetic resonance imaging (MRI) of the affected individuals is considered a clinical feature specific to the disease. MRI brain demonstrated iron accumulation in the brain through a low signal within a hyperintense region in the globus pallidus area on T2-weighted image in both the atypical and classic form.^[3,5]^ Other types of neurodegeneration due to brain iron accumulation that are not related to the *PANK2* gene mutations usually do not exhibit the “eye of the tiger” sign.^[6]^ Thus, a diagnosis is made based on the clinical history and MRI imaging. Recently, with the advantages of next-generation sequencing, the application of whole exome sequencing (WES) has been widely used to improve efficiency in diagnosis.^[7]^

The *PANK2* gene (NM_153638.4, OMIM#606157), encoding the enzyme that catalyzed the phosphorylation of pantothenate (vitamin B5) in the bioavailability of coenzyme A (CoA), was identified as the causative gene of the PKAN disease. The *PANK2* gene, present in the mitochondria and the nucleus, is located on chromosome 20p13 and spans approximately 34 kb with 7 exons.^[3,8–10]^ The *PANK2* gene encodes an important regulatory enzyme (consisting of 571-amino acids) among four pantothenate kinase enzymes that are targeted to mitochondria of CoA biosynthesis. The formation of CoA is essential for energy metabolism, fatty acid synthesis, degradation, and synthesis of many neurotransmitters as well as glutathione, an antioxidant that prevents cell damage caused by reactive oxygen species. The deficiency of CoA in energy production can lead to forming of reactive oxygen species free radicals and damaging cells.^[11]^ Inactivation of the PANK2 protein leads to the accumulation of cysteine-containing substrates and iron-induced lipid peroxidation which is responsible for PKAN. In addition, cysteine is a substrate of the PANK2 enzyme and may play a key role in iron accumulation, therefore in PKAN patients, there is an increase of cysteine and chelate leading to the accumulation of iron in the brain.^[11,12]^ It is thought that excessive accumulation of cysteine in the globus pallidus region results in decreasing pantothenate, causing iron chelation and oxidative damage to basal ganglia. However, how a defect in mitochondrial CoA synthesis leads to iron accumulation and the specific PKAN phenotype remains unclear.

NBIA caused by PANK2 mutations has been reported previously in Asian ethnic groups, for example, Chinese, Korean, Indian, Iranian, Taiwan, and Thailand.^[8,13–24]^ In this article, we report the first PKAN patient in Vietnam due to the PANK2 mutations.

## 2. Case presentation

### 2.1. Clinical presentation

**Figure 1.**
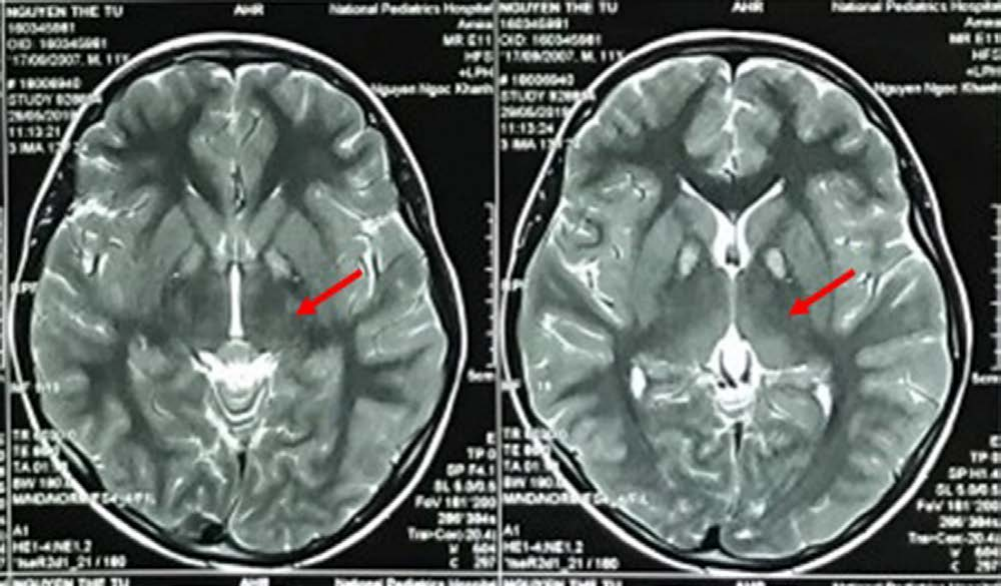
T2 weighted MR imaging in the patient with the “eye of the tiger” sign, which is a characteristic feature of pantothenate kinase-associated neurodegeneration, showed an area of marked hypointensity in the globus pallidus.

The patient is a 12-year-old boy who presented with a three-year history of muscle weakness, gait disturbance, dysarthria, and dysphagia since the age of 10. Other clinical symptoms observed include difficulty walking, easy falling, skinny body, reduced vision, difficulty holding a pen, and difficulty chewing and swallowing. Evidence of iron accumulation in the brain as the “eye of the tiger” sign detected on the MRI showing a hyperintensity in the globus pallidus region on T2-weighted images (Fig. 1). A low signal indicates iron deposition and a high signal represents tissue edema or reactive changes in the globus pallidus region observed in MRI image. The biochemical test index, such as serum ammonia (44.4 mg/dL), glucose (5.5 mmol/L), GOT (Glutamic oxaloacetic transaminase) (43.1 UI/L), and ALT (Alanine aminotransferase) (25.1 UI/L) are all within the normal range. Lactate (1.07 mmol/L) levels are higher than the normal range and levels of urine ketones are negative.

### 2.2. Ethics statement

The study was approved by the Ethics Committee of the Institute of Genome Research (Approval No. 01-2022/NCHG-HDDD). The consent form was written by the patient’s parents for the publication of any potentially identifiable images or data included in the article.

### 2.3. Molecular investigation

**Figure 2.**
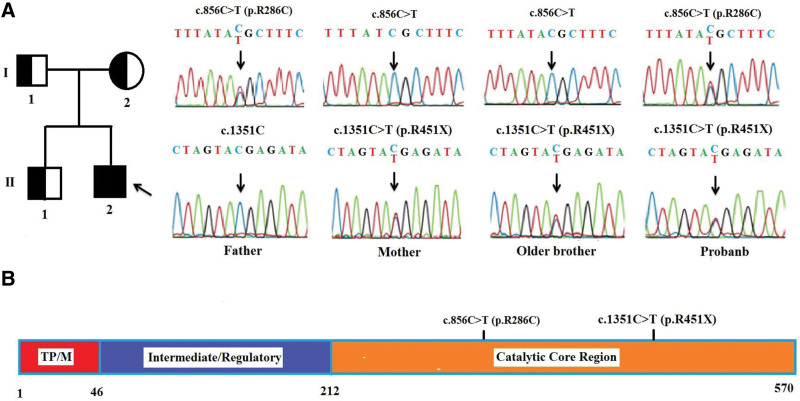
(A) Pedigree and the Sanger sequencing results of the members of the patient’s family. The results show that a heterozygous mutation (c.856C > T, p.Arg286Cys) in the *PANK2* gene is detected in the patient and his father, and another heterozygous mutation (c.1351C > T, p.Arg451Ter) is found in the patient, his mother, and his older brother. (B) Position of the mutations in the structure model of the *PANK2* protein. *PANK2* = pantothenate kinase 2.

Peripheral blood of the patient and the unaffected members of his family were collected for molecular analysis. Genomic DNA was extracted from samples using a Qiagen DNA blood mini kit (QIAGEN, Hilden, Germany) following manufacturer guidelines. The DNA was used for WES on the Illumina platform (Illumina Inc.). The GRCh38 human genome was used as reference data for mapping the paired-end reads by BWA0.7.17 software.^[25]^ The post-alignment process of data includes creating indexes, marking, removed repeated reads on the alignment bam file used by the Picard tool (https://broadinstitute.github.io/picard/command-line-overview.html). The GATK package version 4.1 was used for calling variants.^[26]^ Sanger sequencing was done for validating these 2 mutations in the patient and the members of his family. PCR amplification was carried out on an Eppendorf Mastercycler EP gradient (USA Scientific, Inc). PCR products were sequenced on ABI PRISM 3500 Genetic Analyzer machine (Thermo Fisher Scientific Inc.). The sequencing data were analyzed using BioEdit 7.2.5 software. Two mutations (c.856C > T, p.Arg286Cys and c.1351C > T, p.Arg451Ter) have been detected in the *PANK2* gene (NM_153638.4) in a compound heterogeneous status. According to ACMG guidance (The American College of Medical Genetics and Genomics), both mutations are reported as pathogenic. These mutations (c.856C > T, p.Arg286Cys and c.1351C > T, p.Arg451Ter) have been registered in the SNP database (https://www.ncbi.nlm.nih.gov/snp/) with accession number rs137852962, rs1250997630 and ClinVar database (https://www.ncbi.nlm.nih.gov/clinvar/) with accession number VCV000004551.3, VCV000667397.6, respectively. The heterozygous mutation c.856C > T, p.Arg286Cys was found in the father of the patient, and the heterozygous mutation c.1351C > T, p.Arg451Ter was found in the mother and old brother of the patient (Fig. 2A).

## 3. Discussion

Pantothenate-associated neurodegeneration (PKAN) is a form of the inherited progressive neurological disease called NBIA disorder, a term introduced by Hayflick et al in 2003,^[3]^ included all neurological disorders with progressive extrapyramidal symptoms and intellectual disability. The pathogenesis of the NBIA disorders as well as the role of iron in the causal of neurodegeneration has not been elucidated yet.^[2]^ Diagnosis of PKAN is based on clinical characteristics and anatomopathological findings. The classic form accounts for 75% of all PKAN cases with a mean age of onset at 6 years (range from 6 months to 12 years), the remainder is the atypical form with a mean age of onset of 14 years (range from 1 to 28 years). In this study, symptoms of gait disturbance, dystonia, dysarthria, dysphagia, reduced vision, and difficulty holding, chewing, and swallowing were observed in the first typical case of PKAN in Vietnam. However, several other features such as choreoathetosis, spasticity, Parkinson, tremor, and increased deep tendon reflexes that were reported in Asian patients but not in the Vietnamese patient. These features were observed more frequently in Asian patients than in Caucasians. Asian patients also have less complex presentations and a lower prevalence of pyramidal signs, mental impairment, and parkinsonism than Caucasians.^[8]^

Diagnosis is made based on the clinical history and MRI results (the “eye of the tiger” sign demonstrated iron accumulation in the brain), and recently, WES has been used to reduce the time and improve diagnosis efficiency.^[7]^ The *PANK2* gene (NM_153638.4, OMIM#606157) was identified as the causative gene of the PKAN disease. PANK2 is a homodimer with each monomer including 2 conserved domains (domain A, aa 208–356, and B, aa 357–566) and the long N-terminal region (with 200 amino acids). Dimerization is through long α-helices (aa 486–513) and 2 reaction regions on an extended loop (aa 446–471) and another helix (aa 414–426). The ATP binding site is located between domain A (aa 219–221) and domain B (aa 520–522) with active site E338 located in domain A.^[27]^ *PANK2* mutations including insertion, deletion, and frameshift mutations in the *PANK2* gene are linked to early-onset disease. In contrast, the missense mutations had in both early- and late-onset disease.^[3]^ The early-onset patients had missense mutations in the mitochondrial trafficking domain (transit peptide/mitochondrial domain) of PANK2 and showed a decreased mitochondrial membrane potential with severity phenotype more than that from late-onset patients with mutations in the other 2 domains.^[18]^

Among the 2 heterozygous mutations found in our patient, c.1351C > T (p.Arg451Ter) mutation (exon 4) has been reported in Taiwan and Korean patients.^[8,21,28]^ The mutation of c.856C > T (p.Arg286Cys) in exon 2 has been previously reported in German patients with hand and gait dystonia^[29]^ and in Chinese patients who presented the symptoms of inarticulation, bradykinesia, gait instability, hyperextension.^[30]^ These mutations locate in the helices of the catalytic core region in the PANK2 protein. The mutation c.856C > T (p.Arg286Cys) locates at the β-strand domain (aa 278–288) and the mutation c.1351C > T (p.Arg451Ter) locates at the ATPase nucleotide binding site domain (aa 442–483). These sites are important for the activation of the PANK2 protein (Fig. 2B). The compound heterozygous mutations found in a Vietnamese patient could result in PANK2 deficiency, possibly leading to PKAN.

## 4. Conclusions

In this study, we performed WES analysis and identified a compound heterozygous mutation (c.856C > T, p.Arg286Cys and c.1351C > T, p.Arg451Ter) in the *PANK2* gene in the first Vietnamese patient with PKAN. The patient’s case marked an important milestone for the first case of the disease in Vietnam. The results provide evidence for a definitive diagnosis to orientate the treatments and genetic counseling for the patient.

## Acknowledgments

We would like to thank the financial support by Vietnam Academy of Science and Technology for Institute of Genome Research, KHCBSS.01/22-24. We would like to express our deep gratitude to the patient who participated in this study.

## Author contributions

**Conceptualization:** Van Khanh Tran.

**Data curation:** Chi Dung Vu, Hai Anh Tran.

**Formal analysis:** Nguyen Thi Kim Lien.

**Investigation:** Chi Dung Vu, Hai Anh Tran.

**Methodology:** Chi Dung Vu, Hai Anh Tran, Nguyen Van Tung, Nguyen Ngoc Lan.

**Software:** Nguyen Van Tung, Nguyen Ngoc Lan.

**Supervision:** Nguyen Huy Hoang.

**Visualization:** Van Khanh Tran.

**Writing – original draft:** Van Khanh Tran, Nguyen Thi Kim Lien.

**Writing – review & editing:** Van Khanh Tran, Nguyen Thi Kim Lien, Huy Thinh Tran, Nguyen Huy Hoang.
